# Enhancing research culture in academia: a spotlight on early career researchers

**DOI:** 10.1186/s12868-023-00816-1

**Published:** 2023-08-29

**Authors:** Kim I Chisholm, Mattéa J. Finelli

**Affiliations:** 1 Pain Centre Versus Arthritis, School of Life Sciences, University of Nottingham, Nottingham, UK; 2 School of Medicine, Biodiscovery Institute, University of Nottingham, Nottingham, UK

## Abstract

This editorial highlights common challenges faced by early career researchers (ECRs) who play a crucial role in our research community. We propose that enhancing the experiences of ECRs will yield benefits to the entire scientific community and we give practical suggestions on how such improvements may be achieved.

## Main text

The term “early career researcher” (ECR) includes a broad range of definitions that vary across countries, universities, and even departments and individuals. In general, an ECR refers to a scientist who is in the initial years following the completion of their PhD. But given the variety of career paths in science, a time-based definition seems inadequate. Instead, we support a move towards a definition which takes into account career stage, funding income and level of independence. With this in mind, let’s meet Alex:

Alex is a typical ECR (a fictitious character that embodies the combined experiences of many ECRs). Besides driving their own research project, Alex also takes on the responsibilities of training and supervising students in the laboratory, managing lab consumables (including the important task of organizing lab birthday cakes), generating data to support theirs and others’ publications, and actively contributing to grant writing. Passionate about Science and deeply invested in their project, Alex finds fulfilment in the diverse range of tasks they undertake, including teaching, mentoring, conducting experiments, writing, and presenting. But, at times, despite their substantial efforts, long hours, and unwavering dedication, Alex feels a lack of recognition, as if their hard work and contributions are taken for granted and not truly acknowledged.

Despite Alex’s objectively good performance, publishing in suitable journals, delivering impressive presentations, fostering successful collaborations, and effectively training others, a persistent sense of inadequacy remains. In every presentation, meeting, and interview Alex is afraid of being exposed as an imposter. Alex strives to remain transparent about these anxieties and realises that most other ECRs feel the same. But the feeling of inadequacy remains deeply entrenched, seemingly impervious to any evidence to the contrary.

As Alex’s postdoctoral years progress, the burden of job insecurity weighs heavily upon them. The constant cycle of short-term contracts and ongoing struggles to secure funding intensify. These challenges are further exacerbated now that Alex has a young family. A critical juncture has arrived for them, a phase experienced by many ECRs, where the need for continuity in academia becomes a pressing concern and has the potential to end the careers of many promising ECRs.

As a research community, we must acknowledge the invaluable role of ECRs, like Alex, in driving scientific progress and, as such, we must prioritize the well-being and professional growth of ECRs. They make up the majority of our research workforce [1] and, like Alex, bear the brunt of research activities, training, and writing, actively participating in every aspect of research creation, implementation, and output. Creating a better work environment and culture for them, one that is more supportive, rewarding, and secure, will lead to better Science. But where do we start? Here are some suggestions:

ECR-Driven Initiatives: ECRs themselves, with support structures from senior colleagues, can play an active role in shaping their experiences:

1. Support Networks: ECRs could be given the necessary time and resources to establish and maintain supportive networks with their peers. Formalised mentoring arrangements in every institution where funding and time allocation are available, would help support these initiatives.
2. Independent Funding: Many promising ECRs, like Alex, delay applying for independent funding longer than necessary. It is common to see researchers waiting for that one significant paper, those seemingly key results or the ideal funding call, before taking the leap to start an independent application. Our advice is to start the process even before everything is perfectly aligned. This approach offers several benefits: It helps identify gaps that need to be addressed, overcomes the inertia that can seem insurmountable, encourages ECRs to carve out a research niche for themselves, provides a fresh perspective on their research, and offers valuable practice in this very new skill.
3. Identifying Opportunities: ECRs can proactively explore avenues to broaden their experiences and expand their presence within the scientific community. They may, for example, pursue positions in scientific committees locally or in academic societies. Another option is to reach out to journals and offer to volunteer as a reviewer. These and similar initiatives not only offer firsthand experience in future career roles but also help expand networks and foster new peer and mentoring relationships. Institutions and senior colleagues would be well placed to encourage and value these contributions to an ECRs career development and to the scientific community as a whole.

Support from Senior Colleagues: More experienced researchers can catalyse positive changes by:

1. Fostering Official Roles of Independence: Senior colleagues can offer ECRs opportunities to publish as senior authors, involve them as co-investigators in grant applications, and invite them to join PhD supervisory teams.
2. Providing Leadership Roles: Established PIs can delegate some official leadership roles (e.g., in learned societies, internal funding panels, committees) to ECRs, with appropriate support and training. This would empower ECRs and even relieve the burden on more established colleagues who may be stretched by their existing leadership roles.
3. Facilitating Access to Pilot Data: To gain independence, many ECRs will need access to pilot data. However, in many cases they may first need independence to have the time and resources to collect such pilot data – a Catch-22. To break this cycle, supportive measures could be put in place to allow ECRs access to existing pilot data or the time/resources to collect these themselves.
4. Gifting independence: Established colleagues could choose to ‘gift’ aspects of research projects to the ECRs who had led them, when these ECRs pursue independence. By doing so, they enable ECRs to establish their own independent research niche, free from direct competition with their larger, better-funded postdoc lab [2].

With this in mind, we want this special issue to be a place where successful research driven by ECRs can be showcased and celebrated. We invite ECRs in the Neuroscience field to submit their manuscripts to this special issue, as first, middle, or senior authors.

## Data Availability

N/A.

## List of abbreviations

ECR: Early career researcher
